# Efficient Serial and Parallel Algorithms for Selection of Unique Oligos in EST Databases

**DOI:** 10.1155/2013/793130

**Published:** 2013-04-08

**Authors:** Manrique Mata-Montero, Nabil Shalaby, Bradley Sheppard

**Affiliations:** ^1^Department of Computer Science, Memorial University, Canada; ^2^Department of Mathematics and Statistics, Memorial University, Canada

## Abstract

Obtaining unique oligos from an EST database is a problem of great importance in bioinformatics, particularly in the discovery of new genes and the mapping of the human genome. Many algorithms have been developed to find unique oligos, many of which are much less time consuming than the traditional brute force approach. An algorithm was presented by Zheng et al. (2004) which finds the solution of the unique oligos search problem efficiently. We implement this algorithm as well as several new algorithms based on some theorems included in this paper. We demonstrate how, with these new algorithms, we can obtain unique oligos much faster than with previous ones. We parallelize these new algorithms to further improve the time of finding unique oligos. All algorithms are run on ESTs obtained from a Barley EST database.

## 1. Introduction

Expressed Sequence Tags (or ESTs) are fragments of DNA that are about 200–800 bases long generated from the sequencing of complementary DNA. ESTs have many applications. They were used in the Human Genome Project in the discovery of new genes and are often used in the mapping of genomic libraries. They can be used to infer functions of newly discovered genes based on comparison to known genes [1].

An oligonucleotide (or oligo) is a subsequence of an EST. Oligos are short, since they are typically no longer than 50 nucleotide bases. Oligos are often referred to in the context of their length by adding the suffix “mer”. For example, an oligo of length 9 would be referred to as a 9-mer. The importance of oligos in relation to EST databases is quite significant. An oligo that is unique in an EST database serves as a representative of its EST sequence. The oligonucleotides (or simply oligos) contained in these EST databases have applications in many areas such as PCR primer design, microarrays, and probing genomic libraries [2–4].

In this paper we will improve on the algorithms presented in [2] to solve the *unique oligos search* problem. This problem requires us to determine all oligos that appear in one EST sequence but not in any of the others. In addition, we will consider two oligos to be virtually identical if they fall within a certain number of mismatches from each other. In the appendix we include all the algorithms used and developed in this paper.

## 2. The Unique Oligos Search Problem

In this paper we use the notation HD(*x*, *y*) to denote the Hamming Distance between the strings *x* and *y*. Given an EST database *D* = {*x*_1_, *x*_2_,…, *x*_*k*_}, where *x*_*i*_ is a string over the alphabet {A, C, G, G}, integers *d* and *l*, and *l*-mer *y*, we say that *y* occurs approximately in *D* if there exists a substring *z* of some EST *x*_*i*_ such that HD(*y*, *z*) ≤ *d*. We also say that an *m*-mutant list of a string *s* is a list of all possible strings, s*, of length |*s*| over the alphabet {A, C, G, T} such that HD(*s*, s*) ≤ *m*. Such a string s* is referred to as an *m*-mutant of *s*. A unique oligo of *D* is defined as an *l*-mer *u* such that *u* occurs exactly in one EST and does not occur approximately in any other EST. The unique oligos search problem is the problem of finding all unique oligos in an EST database.

Many algorithms have been presented to solve this problem [5, 6]. The algorithm presented in [2] relies on an observation that if two *l*-mers agree within a specific Hamming Distance, then they must share a certain substring. These observations are presented in this paper as theorems.

**Theorem 1**

Suppose one has two *l*-mers *l*_1_ and *l*_2_ such that *HD*(*l*_1_, *l*_2_) ≤ *d*. If one divides them both into ⌊*d*/2⌋ + 1 substrings, *l*_1_^1^ *l*_1_^2^ ⋯ *l*_1_^⌊*d*/2⌋+1^ and *l*_2_^1^ *l*_2_^2^ ⋯ *l*_2_^⌊*d*/2⌋+1^, and each *l*_*j*_^*i*^, except possibly *l*_*j*_^⌊*d*/2⌋+1^, has length ⌈*l*/(⌊*d*/2⌋ + 1)⌉, then there exists at least one *i*_0_ ∈ {1,2,…, ⌊*d*/2⌋ + 1}, such that HD(*l*_1_^*i*_0_^, *l*_2_^*i*_0_^) ≤ 1.

**Proof**

Suppose by contradiction that for any *i* ∈ {1,2,…, ⌊*d*/2⌋ + 1}, *l*_1_^*i*^ and *l*_2_^*i*^ have at least 2 mismatches. Then HD(*l*_1_, *l*_2_) ≥ *d* + 2 which is a contradiction to the fact that HD(*l*_1_, *l*_2_) ≤ *d*.

Using this observation, an algorithm was presented in [2] which solves the unique oligos search problem in time *O*((*l* − *q*)*qr* ^2^4^*q*^). The algorithm can be thought of as a two-phase method. In the first phase we record the position of each *q*-mer in the database into a hash table of size 4^*q*^. We do so in such a way that for each *q*-mer *x* over the alphabet {A, C, G, T} we have that *hashtable*[*hashfunction*[*x*]] = {{*s*_1_, *p*_1_}, {*s*_2_, *p*_2_},…, {*s*_*n*_, *p*_*n*_}} whereby *s*_*i*_ is an EST sequence, *p*_*i*_ is the position of *x* within that sequence, and *n* is the number of occurrences of *x* in the database. In the second phase, we extend every pair of identical *q*-mers into *l*-mers and compare these *l*-mers for nonuniqueness. We also do the same for pairs that have a Hamming Distance of 1. If they are nonunique, we mark them accordingly.  Theorem 1 guarantees that if an *l*-mer is nonunique, then it must share a *q*-mer substring that differs by at most one character with another *q*-mer substring from another *l*-mer. Hence, if an *l*-mer is nonunique, it will be marked during phase two.

Assuming there are *n* symbols in our EST database, the filing of the *q*-mers into the hash table takes time Θ(*qn*). In phase two, we assume that the distribution of *q*-mers in the database is uniform; in other words, that each table contains *r* ≈ *n*/4^*q*^ entries. Thus we have *O*(*r* ^2^) comparisons within each table entry. Each *q*-mer also has a 1-mutant list of size 3*q*, so, we have *O*(*qr* ^2^) comparisons for each entry in the table. Also, the time required to extend each pair of *q*-mers to *l*-mers is 2(*l* − *q* + 1). Given that we have 4^*q*^ entries in the hash table, we have a total time complexity of

(1) O((l−q)qr24q)=O((l−q)q(n4q)24q)=O((l−q)qn24q),

where

(2) $$\begin{array}{c} q=\frac{l}{\left\lfloor d/2\right\rfloor +1}. \end{array}$$

In [7], several variations of Theorem 1 are presented. We can use these theorems to generate similar algorithms with slightly different time complexities.

**Theorem 2**

Suppose one has two *l*-mers *l*_1_ and *l*_2_ such that HD(*l*_1_, *l*_2_) ≤ *d*. If one divides them both into *d* + 1 substrings, *l*_1_^1^ *l*_1_^2^ ⋯ *l*_1_^*d*+1^ and *l*_2_^1^ *l*_2_^2^ ⋯ *l*_2_^*d*+1^, and each *l*_*j*_^*i*^, except possibly *l*_*j*_^*d*+1^, has length ⌈*l*/(*d* + 1)⌉, then there exists at least one *i*_0_ ∈ {1,2,…, *d* + 1}, such that *l*_1_^*i*_0_^ = *l*_2_^*i*_0_^.

**Proof**

Suppose by contradiction that we cannot find any *i*_0_ ∈ {1,2,…, *d* + 1} such that *l*_1_^*i*_0_^ = *l*_2_^*i*_0_^. Then there exists at least one mismatch between *l*_1_^*i*^ and *l*_2_^*i*^ for each *i* ∈ {1,2,…, *d* + 1}, and thus we have at least *d* + 1 mismatches which contradicts the fact that HD(*l*_1_, *l*_2_) ≤ *d*.

Based on Theorem 2 we can design a second algorithm that works in a similar way to Algorithm 1. The major difference between these algorithms is that in Algorithm 2 we are not required to do comparisons with each hash table entries mutant list. This means we have *O*(*r* ^2^) comparisons within each table entry which yields a total time complexity of

(3) O((l−q)r24q)=O((l−q)(n4q)24q)=O((l−q)n24q),

where

(4) $$\begin{array}{c} q=\frac{l}{d+1}. \end{array}$$

A third theorem was also briefly mentioned [7]; however, it was not implemented in an algorithm. We use this theorem to create a third algorithm to solve the unique oligos search problem.

**Theorem 3**

Suppose one has two *l*-mers *l*_1_ and *l*_2_ such that HD(*l*_1_, *l*_2_) ≤ *d*. If one divides them both into ⌊*d*/3⌋ + 1 substrings, *l*_1_^1^ *l*_1_^2^ ⋯ *l*_1_^⌊*d*/3⌋+1^ and *l*_2_^1^ *l*_2_^2^ ⋯ *l*_2_^⌊*d*/3⌋+1^, and each *l*_*j*_^*i*^, except possibly *l*_*j*_^⌊*d*/3⌋+1^, has length ⌈*l*/(⌊*d*/3⌋ + 1)⌉, then there exists at least one *i*_0_ ∈ {1,2,…, ⌊*d*/3⌋ + 1}, such that HD(*l*_1_^*i*_0_^, *l*_2_^*i*_0_^) ≤ 2.

**Proof**

Suppose by contradiction that for any *i* ∈ {1,2,…, ⌊*d*/3⌋ + 1}, *l*_1_^*i*^ and *l*_2_^*i*^ have at least 3 mismatches. Then HD(*l*_1_, *l*_2_) ≥ *d* + 3 which is a contradiction to the fact that HD(*l*_1_, *l*_2_) ≤ 2.

The algorithm is somewhat similar to Algorithm 1. The main difference is that we compare every *q*-mer to *q*-mers in its corresponding 2-mutant list, rather than its 1-mutant list. Each *q*-mer has $9\left(\begin{array}{c} q\\ 2 \end{array}\right)+3q=9q(q-1)/2+3q$ 2-mutants, so we have *O*(*q* ^2^ *r* ^2^) comparisons for each entry in the hash table yielding a total time complexity of

(5) O((l−q)q2r24q)=O((l−q)q2(n4q)24q)=O((l−q)q2n24q),

where

(6) $$\begin{array}{c} q=\frac{l}{\left\lfloor d/3\right\rfloor +1}. \end{array}$$

It is important to note the 4^*q*^ term in the denominator of our time complexity expressions. Since this term is exponential, it will have the largest impact on the time taken to run our algorithms. Based on this observation, we expect Algorithm 3 to run the fastest, followed by Algorithm 1 and then Algorithm 2.

## 3. Implementation

We implement these algorithms using C on a machine with 12 Intel Core i7 CPU 80 @ 3.33 GHz processors and 12 GB of memory. The datasets we use in this implementation are Barley ESTs taken from the genetic software HarvEST by Steve Wanamaker and Timothy Close of the University of California, Riverside (http://harvest.ucr.edu/). We use two different EST databases, one with 78 ESTs and another with 2838. In our experiments we search for oligos of lengths 27 and 28 since they are common lengths for oligonucleotides. As we increase the size of the database, we see that Algorithm 3 is the most efficient as anticipated (data shown in Tables 1 and 2).

One important thing to note about all of these algorithms is the fact that the main portion of them is a for loop which iterates through each index of the hash table. It is also obvious that loop iterations are independent of each other. These two factors make the algorithms perfect candidates for parallelism. Rather than process the hash table one index at a time, our parallel algorithms process groups of indices simultaneously. Ignoring the communication between processors, our algorithms optimally parallelize our three serial algorithms.

There are many APIs in different programming languages that aid in the task of parallel programming. Some examples of this in the C programming language are OpenMP and POSIX Pthreads. OpenMP allows one to easily parallelize a C program amongst multiple cores of a multicore machine [8]. OpenMP also has an extension called Cluster OpenMP which allows one to parallelize across multiple machines in a computing cluster.

A new trend in parallel programming is in the use of GPUs. GPUs are the processing units inside computers graphics card. C has several APIs which allow one to carry out GPU programming. The two such APIs are OpenCL and CUDA [9, 10].

In the second implementation of our algorithms we use OpenMP to parallelize our algorithms throughout the 12 cores of our machine. We can easily see that we achieve near optimal parallelization with our parallel algorithms; that is, the time taken by the parallel algorithms is approximately that of the serial algorithms divided by the number of processors.

## 4. Conclusion

In this paper we used three algorithms to solve the unique oligos search problem which are extensions of the algorithm presented in [2]. We observed that we can achieve a significant performance improvement by parallelizing our algorithms. We can also see that Algorithm 3 yields the best results for larger databases. For smaller databases, however, the time difference between each pair of algorithms is negligible, but results in Algorithm 3 being the slowest, and this is due to the time required to compute the mismatches of each *q*-mer. Other algorithms can be obtained by setting *q* to different values. See Algorithms 1, 2, 3, 4, 5, 6, 7, and 8. 

## Figures and Tables

**Algorithm 1:**
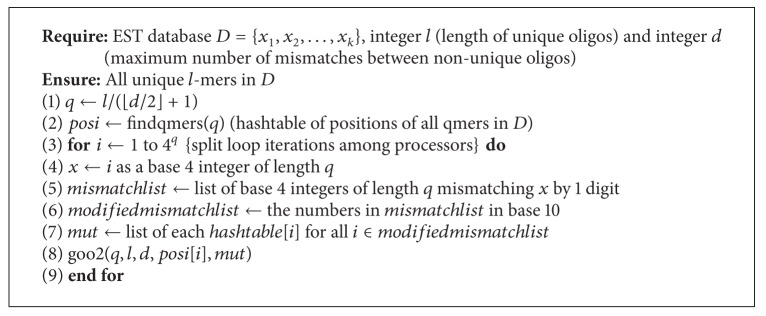
Algorithm for the unique oligos problem.

**Algorithm 2:**
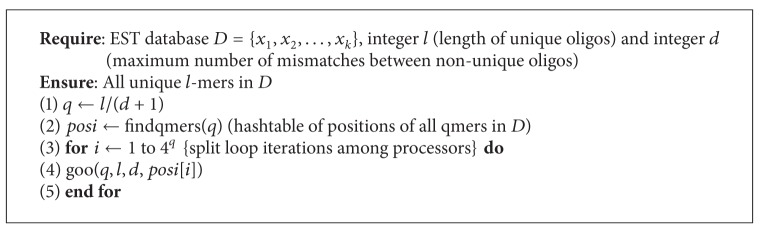
Algorithm for the unique oligos problem.

**Algorithm 3:**
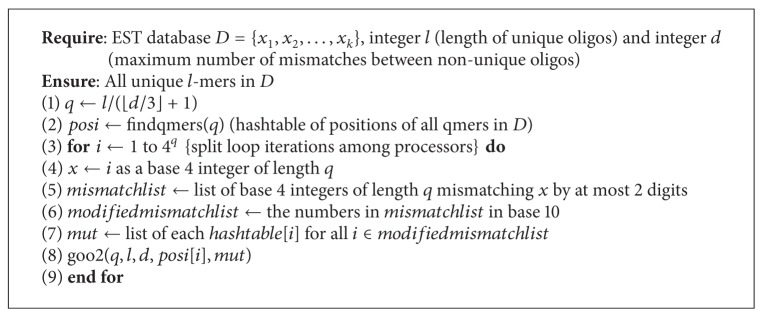
Algorithm for the unique oligos problem.

**Algorithm 4:**
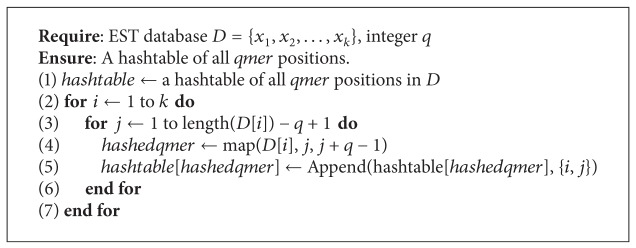
Findqmers (*q*).

**Algorithm 5:**
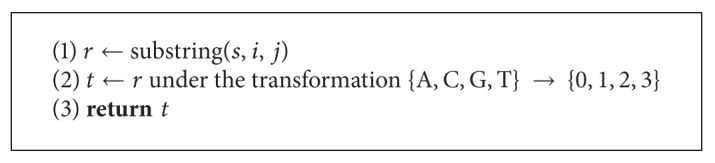
Map (string *s*, *i*, *j*).

**Algorithm 6:**
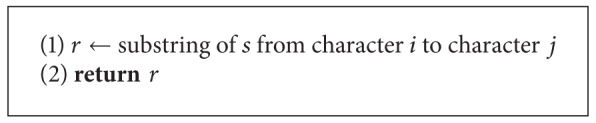
Substring (string *s*, *i*, *j*).

**Algorithm 7:**
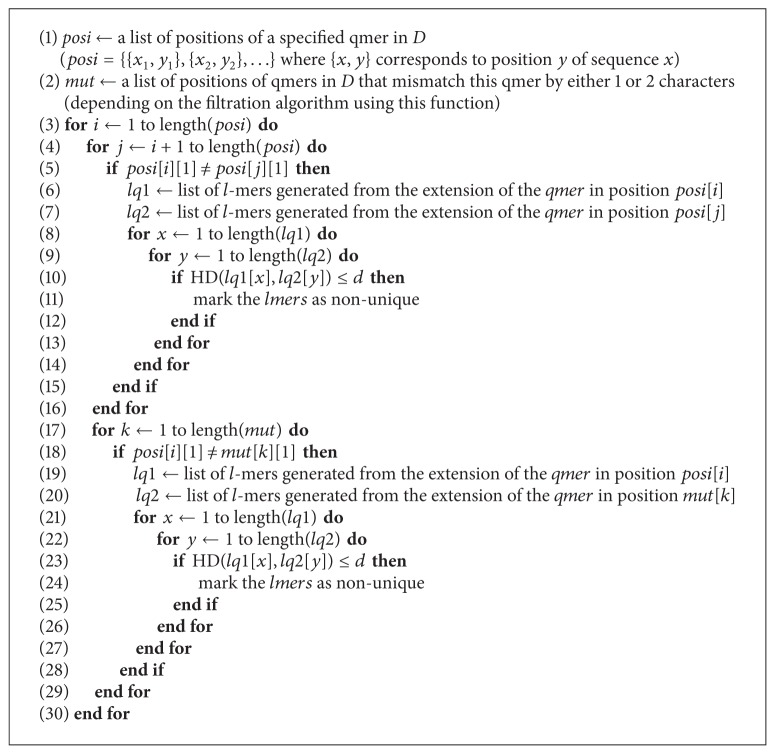
goo2(*q*, *l*, *d*, *posi*, *mut*).

**Algorithm 8:**
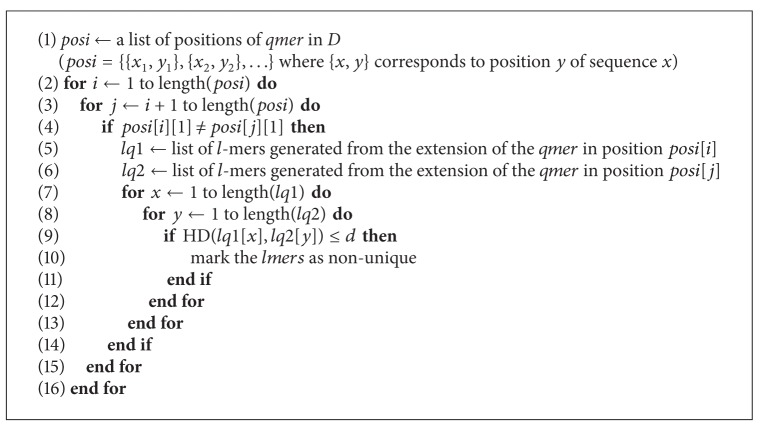
goo(*q*, *l*, *d*, *posi*).

**Table 1:** Results of serial algorithms.

Algorithm	*l*	*d*	*q*	Dataset	Time taken (secs)	Non-unique oligos
Algorithm 2	28	6	4	1 (78 ESTs)	163	46,469
Algorithm 1	28	6	7	1 (78 ESTs)	131	46,469
Algorithm 3	27	6	9	1 (78 ESTs)	231	46,564
Algorithm 2	28	6	4	2 (2838 ESTs)	197, 500	1,611,241
Algorithm 1	28	6	7	2 (2838 ESTs)	117, 714	1,611,241
Algorithm 3	27	6	9	2 (2838 ESTs)	94, 317	1,614,235

**Table 2:** Results of parallel algorithms on 12 processors.

Algorithm	*l*	*d*	*q*	Dataset	Time taken (secs)	Non-unique oligos
Algorithm 2	28	6	4	1 (78 ESTs)	33	46,469
Algorithm 1	28	6	7	1 (78 ESTs)	29	46,469
Algorithm 3	27	6	9	1 (78 ESTs)	66	46,564
Algorithm 2	28	6	4	2 (2838 ESTs)	40, 420	1,611,241
Algorithm 1	28	6	7	2 (2838 ESTs)	22, 848	1,611,241
Algorithm 1	27	6	9	2 (2838 ESTs)	18, 375	1,614,235
